# Twin pregnancy and postpartum haemorrhage: a systematic review and meta-analysis

**DOI:** 10.1186/s12884-024-06798-0

**Published:** 2024-10-04

**Authors:** Fatma A. M. Abdulsalam, Natalie E. Bourdakos, James W. F. Burns, Zoe Y. Zervides, Nathanael Q. E. Yap, Maamoun Adra, Hayato Nakanishi, Christian A. Than, Francis A. Chervenak, Sir Sabaratnam Arulkumaran

**Affiliations:** 1 Milton Keynes University Hospital, Milton Keynes, MK6 5LD UK; 2 George’s University of London, London, SW17 0RE UK; 3 Medical School, University of Nicosia, University of Nicosia, Nicosia, Cyprus; 4 Manchester University NHS Foundation Trust, Cobbett House, Oxford RoadGreater Manchester, Manchester, M13 9WL UK; 5 School of Biomedical Sciences, The University of Queensland, St Lucia, Australia; 6 Lenox Hill Hospital, New York, NY 10075 USA; 7 Donald and Barbara Zucker School of Medicine at Hofstra/Northwell, Hempstead, NY 11549 USA

**Keywords:** Twin pregnancy, Post-partum haemorrhage, PPH

## Abstract

**Background:**

Postpartum haemorrhage (PPH) continues to stand as the primary cause of maternal morbidity and mortality post-delivery, with twin pregnancies carrying a heightened risk of PPH compared to singleton deliveries.

**Objectives:**

To investigate the incidence of primary PPH among twin pregnancies and report on maternal and peripartum characteristics within this population.

**Methods:**

A literature search was conducted using data from PubMed, EMBASE, Cochrane, Scopus, and Web of Science. The search aimed to identify studies concerning mothers with twin pregnancies and postpartum haemorrhage (PPH) from the inception of each respective database to June 8th, 2023. Pooled means and proportions were analyzed using the generic inverse variance method. This review was registered prospectively with PROSPERO (CRD42023427192).

**Results:**

A total of 21 studies involving 23,330 twin pregnant patients were included. Incidence of PPH for vaginal delivery and Caesarean delivery (CS) was found to be 10.9% (95% CI: -0.017, 0.235, I^2^ = 96%) and 27.0% (95% CI: 0.180, 0.359, I^2^ = 99%) respectively. In vitro fertilization (IVF) was the most common conception method at 62.0% (95% CI: 0.448, 0.792, I^2^ = 100%) with 81.1% (95% CI: 0.708, 0.915, I^2^ = 100%) of twins being dichorionic diamniotic.

**Conclusion:**

This meta-analysis demonstrated more than one in ten vaginal deliveries and over one in four cesarean sections result in PPH for twin pregnancies. IVF is the predominant method of conception in this patient group and seems to contribute to subsequent PPH risk in specific mothers. While preliminary, these findings underscore the necessity for further well-designed and high-quality studies to validate these results.

**Supplementary Information:**

The online version contains supplementary material available at 10.1186/s12884-024-06798-0.

## Introduction

Postpartum haemorrhage (PPH), commonly defined as the loss of 500 mL of blood following vaginal birth or 1000 mL following caesarean delivery (CS) [1], is a significant pathological event in obstetrics. PPH is theleading cause of maternal morbidity and mortality after delivery, accounting for approximately 70,000 maternal deaths per year [2]. In 2012, a meta-analysis spanning multiple countries reported incidence of PPH ranged between 7.2% and 25.7%, averaging around 10.8%, with lacking estimates specific for twin deliveries [3]. However, twin pregnancies are estimated to confer a higher risk of PPH and maternal mortality due to PPH than singleton deliveries [4].

The main aetiology behind PPH is uterine atony, which comprises 70–80% of causes [5]. This is due to failure of effective contractions by the uterus after delivery. The predominant risk factor for uterine atony is described as increased distention of the uterus, which in turn impairs myometrial contractility after delivery [6]. Twin pregnancies have been correlated with this phenomenon through the physical occupation of space within the uterus by twin fetuses [4]. Additionally, a larger placental bed, increased maternal blood volume, increased uterine blood flow, greater haemodilution, and anaemia resulting in hyperdynamic circulation, along with increased velocity due to reduced viscosity and reduced anti-fibrinolytic activity, may exacerbate the risk of PPH [7, 8].

The maternal adaptation to pregnancy is evidently compounded in twin pregnancies which leads to several complications. Most concerning is an increase in maternal morbidity, which is 2.5 times higher than singleton pregnancy [9]. Furthermore, twin pregnancies demonstrate an increased risk of hypertensive disorders including gestational hypertension, pre-eclampsia and eclampsia [10, 11]. Disorders surrounding delivery, such as preterm labor and premature rupture of membranes, are also elevated [12]. Twin pregnancies are also well known to increase the risk of placental disorders of placenta previa, placental accreta and placental abruption [10, 13]. The greater likelihood of these placental disorders increases the rates of caesarean delivery which correspondingly increases maternal risk of PPH.

Currently, there is a lack of systematically pooled evidence regarding the incidence of PPH in twin pregnancies. Furthermore, associative features, including comorbidities and neonatal outcomes, of this demographic must be explored. The aim of this paper is to determine the incidence of primary post-partum hemorrhage (PPH) in twin pregnancies and report on maternal and peripartum characteristics within this population.

## Methods

### Search strategy and data sources

In compliance with the Preferred Reporting Items for Systemic Reviews and Meta-analyses (PRISMA) guidelines [14], a comprehensive search of several electronic databases from each database’s inception to June 8th 2023 was conducted. The databases included PubMed, EMBASE, Cochrane, Scopus, and Web of Science. An experienced librarian developed and implemented the search strategy with input from the study's principal investigator. Controlled vocabulary supplemented with keywords was used to search for studies regarding mothers with twin pregnancies. The actual strategy listing all search terms used and how they are combined is available in Supplementary Item 1. This review was registered prospectively with PROSPERO (CRD42023427192). Of note, some of the secondary characteristics listed in the PROSPERO (atony requiring uterotonics, uterine or hypogastric artery ligation, compression sutures, intrauterine balloon tamponade, exploratory laparotomy, ICU admission) were unable to be analyzed within this meta-analysis due to lack of reporting by the included studies. The components of this meta-analysis were organised in accordance with the PRISMA 2020 checklist available in Supplementary Item 2.

### Eligibility criteria and quality assessment

The following inclusion criteria had to be met by eligible studies to be considered for the analysis: 1) be written in the English language; 2) study design of either RCTs, cohort, retrospective, and prospective studies; 3) maternal age greater or equal to 18 years old; 4) reports the following primary outcome: incidence of PPH in twin pregnancies as defined by the Agency for Research Healthcare and Quality, which is the loss of 500 mL of blood following vaginal birth or 1000 mL following caesarean delivery [1]. Triplet or higher order pregnancies, women with pre-existing blood disorders, women taking therapeutic anticoagulants, patients with a fetal death, medically indicated termination of pregnancy, pregnancies with fetal reduction as well as case reports, case series, abstracts, review studies and studies with overlapping patient data were all excluded. Article screening and data extraction were conducted by three independent assessors (NEB, JWFB, ZYZ). Any disagreements were adjudicated by FAMA and discussed with co-authors as necessary. The methodological quality of each study was assessed independently by two authors (JWFB & ZYZ) using the RoB-2 Tool [15] and the ROBINS-I Tool [16]. The results of the quality assessment of all included studies are shown in Supplemental Table 1 and Supplemental Table 2.

### Data extraction

The following maternal characteristics were extracted: mode of conception (natural, in vitro fertilization (IVF), other), chorionicity (dichorionic diamniotic, monochorionic diamniotic, monochorionic monoamniotic), and maternal comorbidities (pre-gestational diabetes, chronic hypertension, anaemia). Post-partum haemorrhage was extracted as > 500 ml of blood loss for vaginal delivery and > 1000 ml of blood loss for CS. The following obstetric outcomes were extracted: gestational diabetes, pre-eclampsia, placental abruption, placenta previa, other placental disorders, preterm pre-labor rupture of membranes (PPROM), pre-labor rupture of membranes (PROM), need for blood transfusion, hysterectomy, decrease in haemoglobin, and prophylactic oxytocin administration. The following neonatal outcomes were extracted: birth weight (g), prevalence of term and pre-term deliveries, APGAR < 7 at 5 min, and neonatal intensive care unit (NICU) admissions.

### Statistical analysis

Means of continuous variables and rates of binary variables were pooled using the generic inverse variance method of DerSimonian, Laird [17]. Proportions underwent logit transformation prior to meta-analysis. The heterogeneity of effect size estimates across the studies was quantified using the Q statistic and the I^2^ index (*P* < 0.10 was considered significant) [18]. A value of I^2^ of 0–25% indicates minimal heterogeneity, 26–50% moderate heterogeneity, and 51–100% substantial heterogeneity. The random-effects model was used [18]. If mean and standard deviation (SD) were unavailable, median was converted to mean using the formulas from the Cochrane Handbook for Systematic Reviews of Interventions [19]. If SD was not available or extractable, the reported mean was omitted from the calculation. Authors were contacted three times to obtain any relevant additional information that was omitted in published articles. Data analysis was performed using Open Meta analyst software (CEBM, Brown University, Providence, Rhode Island, USA).

## Results

### Study selection

The initial literature search of the electronic databases yielded 2,013 studies. After removing duplicates, the articles were screened according to the inclusion and exclusion criteria yielding 399 for full-text review. Finally, 21 studies involving 23,330 pregnant patients met the eligibility criteria and were included in this study [4, 20–39]. The PRISMA flow chart (Supplementary Fig. 1) illustrates the details of the study selection process.

### Risk of bias

Results of the quality assessment of all included studies are included in Supplemental Table 1 and Supplemental Table 2. Randomized control trials judged by ROB – 2 tool were found to be of some concern. However, some of the observational studies judged by ROBINS-1 tool were deemed to be at serious risk of bias [20–22, 25–28, 30, 31, 33–35, 38]. Those studies were found to be of serious concern due to lacking features in domains of randomization and selection of participation. Nonetheless, all studies included adequately reported intended interventions, measuring outcomes and little had missing data.

### Baseline characteristics

The total number of mothers carrying twin pregnancies was 23,330 with a mean gestational age of 36.2 ± 2.3 weeks. Of these, 4,642 (19.9%) underwent vaginal delivery, 14,282 (61.2%) underwent CS, and 153 (0.7%) underwent a combined delivery with vaginal delivery of twin A and CS of twin B. Of note, 4,253 (18.2%) patients did not have delivery status reported. Of those who underwent vaginal delivery, 227 (4.9%) were classified as spontaneous and 23 (0.5%) as operative. The remaining 94.6% are unknown due to lack of reporting in the primary date. Of those who underwent CS, 5,196 (36.4%) were elective and 3,420 (24.0%) were emergency. The remaining 39.7% are unknown due to lack of reporting in the primary data. The baseline characteristics and indications are reported in Table 1.

**Table 1:** Baseline and clinical characteristics

Citation	Study Type	Country	Sample Size (n)	Maternal Age means (SD)	Gestational Age means (SD)	Pre-gestational BMI means (SD)	BMI at delivery means (SD)	Nulliparas (%)	Primiparous (%)	Multiparous (%)	Combined Birth Weight Means (SD)
Anh et al. 2022 [22]	Retrospective	Vietnam	739	30.30 (4.95)	36.37 (2.41)	NR	26.20 (3.03)	NR	NR	NR	2321.71 (433.34)
Cao et al. 2022 [21]	Retrospective	China	3180	31.53 (3.90)	NR	NR	NR	2651 (83.36%)	NR	529 (16.64%)	NR
Chiruvolu et al. 2022 [22]	Retrospective	U.S.	31	27.95 (5.69)	28.07 (2.62)	NR	NR	NR	NR	NR	1108.45 (348.22)
Dayan-Schwartz et al. [23]	Retrospective	Israel	339	31.17 (4.73)	35.77 (3.10)	25.17 (5.40)	NR	NR	NR	NR	2333.83 (614.57)
di Marco et al. 2023 [4]	Retrospective	Italy	707	35.17 (5.96)	NR	NR	NR	468 (66.20%)	189 (26.73%)	50 (7.07%)	2280.06 (391.08)
Drassinower et al.2014 [24]	Prospective	U.S.	1009	29.86 (6.88)	NR	31.97 (6.70)	NR	503 (49.85%)	NR	NR	NR
Huber et al. 2015 [25]	Retrospective	Germany	186	33.03 (5.14)	NR	26.038 (5.05)	NR	NR	98 (52.69%)	88 (47.31%)	NR
Jiang et al. 2021 [26]	Crosssectional	China	3212	30.93 (4.40)	35.57 (2.77)	21.41 (3.18)	NR	NR	2070 (64.45%)	NR	2355.35 (581.82)
Kim et al. 2020 [27]	Retrospective	South Korea	953	34.25 (3.10)	36.05 (2.03)	NR	26.99 (3.22)	833 (87.41%)	120 (12.59%)	NR	NR
Lin et al. 2021 [28]	Retrospective	China	288	29.53 (5.03)	36.01 (2.81)	NR	27.47 (3.75)	NR	NR	NR	NR
Lyu et al. 2022 [29]	Retrospective	China	2472	31.59 (3.81)	35.92 (1.71)	NR	NR	NR	NR	NR	2483.12 (430.94)
Myles et al. 2001 [30]	Retrospective	USA	19	NR	36.30 (4.10)	NR	NR	NR	NR	NR	2370.6 (591)
Santana et al. 2016 [31]	Crosssectional	U.S.	4756	NR	NR	NR	NR	1543 (32.44%)	NR	NR	NR
Sentilhes et al. 2022 [32]	RCT	France	319	33.95 (4.91)	NR	25.00 (6.09)	NR	NR	81 (25.39%)	NR	NR
Seow et al. 2017 [33]	Retrospective	Taiwan	64	34.01 (3.51)	35.20 (2.62)	NR	NR	NR	411 (87.45%)	NR	223.29 (481.83)
Wenckus et al. 2014 [34]	Retrospective	USA	2225	30.43 (6.44)	37.30 (0.95)	NR	32.87 (6.93)	923 (41.48%)	NR	NR	2680.99 (386.78)
Ye et al. 2021 [35]	Observational	China	470	29.49 (4.23)	36.64 (1.56)	21.44 (3.03)	NR	NR	NR	NR	2482.5 (387.37)
Yee at al. 2019 [36]	Observational	U.S.	573	NR	NR	NR	NR	NR	NR	NR	NR
Zhang et al. 2023 [37]	Retrospective	China	372	31.31 (3.85)	NR	NR	NR	287 (77.15%)	NR	85 (22.85%)	NR
Zhu et al. 2016 [38]	Retrospective	China	1071	NR	NR	NR	NR	NR	NR	NR	NR
Zidan et al. 2022 [39]	Retrospective	Israel	345	30.48 (4.99)	36.91 (1.38)	NR	29.45 (4.15)	130 (37.68%)	NR	NR	2499.10 (374.53)

*BMI* Body Mass Index, *NR *Not Reported, *RCT *Randomized Controlled Trial, *SD *Standard Deviation

### Maternal characteristics

#### Conception

**Fig. 1:**
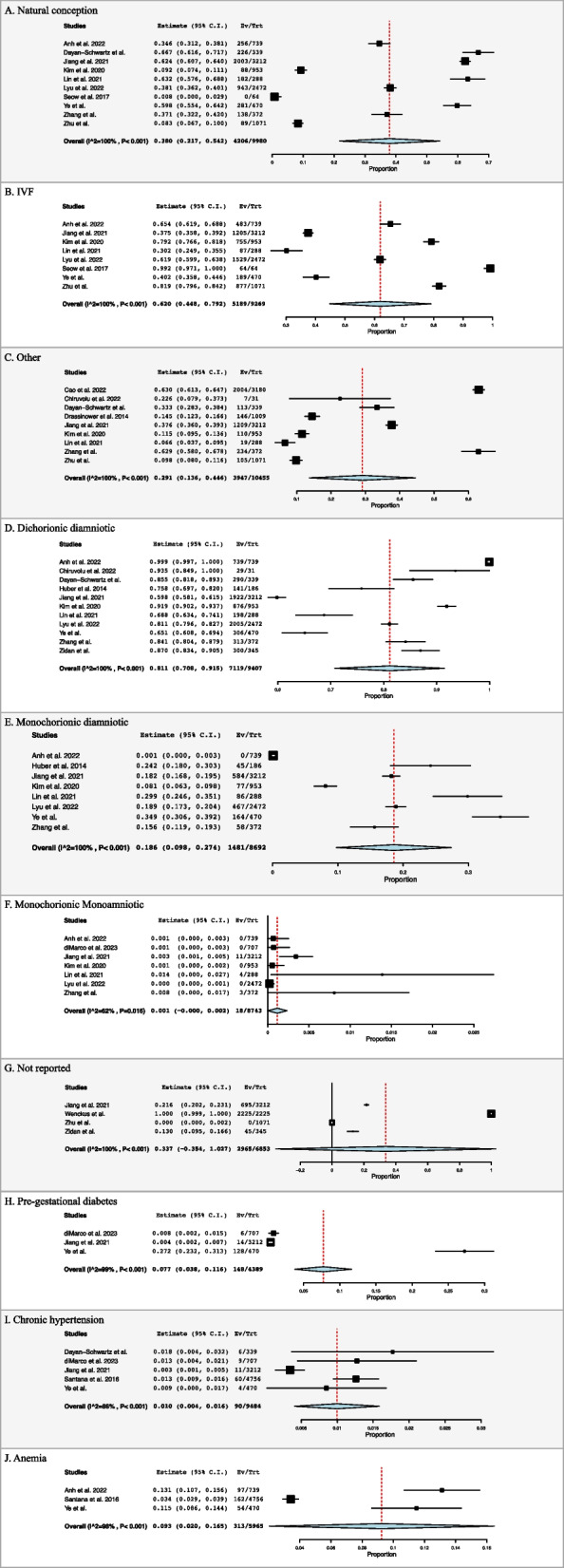
Forest plots for maternal characteristics

Thirteen studies reported on mode of conception [20–24, 26–29, 33, 35, 37, 38]. The prevalence of natural conception, IVF and modes classified as other was found to be 38.0% (95% CI: 0.217, 0.542, I^2^ = 100%, *n* = 4,206), 62.0% (95% CI: 0.448, 0.792, I^2^ = 100%, *n* = 5,189), and 29.1% (95% CI: 0.136, 0.446, I^2^ = 100%, *n* = 3,947) respectively **(**Fig. 1A, B and C**)**.

#### Chorionicity

Twelve studies reported on chorionicity [4, 20, 22, 23, 25–29, 35, 37, 39]. Prevalence of dichorionic diamniotic, monochorionic diamniotic, and monochorionic monoamniotic twins was found to be 81.1% (95% CI: 0.708, 0.915, I^2^ = 100%, *n* = 7119), 18.6% (95% CI: 0.098, 0.274, I^2^ = 100%, *n* = 1481), and 0.1% (95% CI: -0.000, 0.002, I^2^ = 62%, *n* = 18) respectively (Fig. 1D, E and F). Of note four studies reported 2,965 pregnancies as no reported chorionicity 33.7% (95% CI: -0.354, 1.027, I^2^ = 100%, *n* = 2965) (Fig. 1G).

#### Maternal comorbidities

Six studies reported on maternal comorbidities [4, 20, 23, 26, 31, 35]. The prevalence of patients with pre-gestational diabetes was 7.7% (95% CI: 0.038, 0.116, I^2^ = 99%, *n* = 148) (Fig. 1H). Chronic hypertension was prevalent in 1.0% (95% CI: 0.004, 0.016, I^2^ = 86%, *n* = 90) (Fig. 1I). Anaemia was prevalent in 9.3% (95% CI: 0.020, 0.165, I^2^ = 98%, *n* = 313) (Fig. 1J).

### Post-partum haemorrhage

**Fig. 2:**
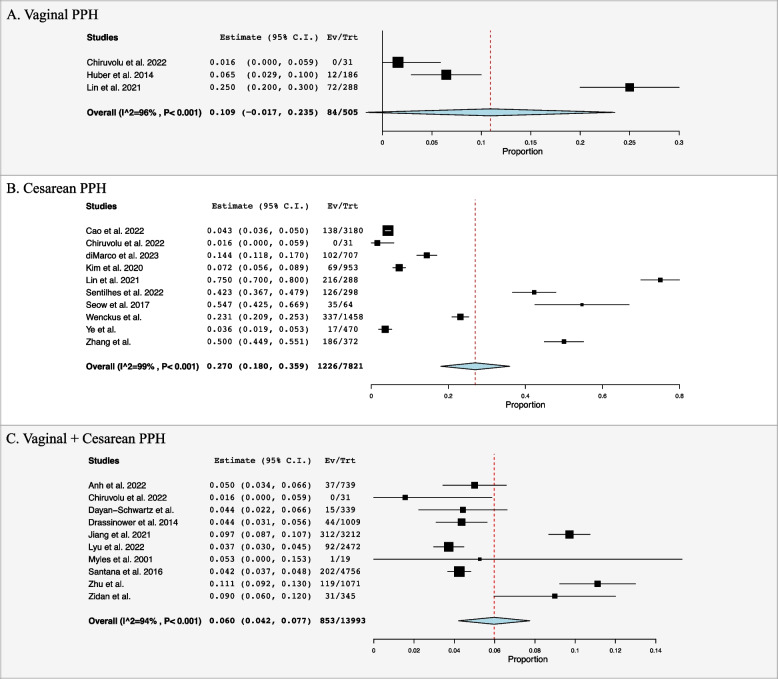
Forest plots for post-partum haemorrhage (PPH)

PPH was reported in twenty studies [4, 20–35, 37–39]. Incidence of PPH for vaginal delivery and CS was found to be 10.9% (95% CI: -0.017, 0.235, I^2^ = 96%, *n* = 84) and 27.0% (95% CI: 0.180, 0.359, I^2^ = 99%, *n* = 1226) respectively (Fig. 2A and B**)**. Of note, ten papers did not differentiate between vaginal and CS delivery when reported PPH, therefore incidence of combined PPH was 6.0% (95% CI: 0.042, 0.077, I^2^ = 94%, *n* = 853) **(**Fig. 2C**)**. Six studies reported on calculated blood loss with a mean loss of 725.7 ± 491.1 mL across both vaginal and CS (Table 2) [27, 32–35, 37].

**Table 2:** Additional Blood Loss Data

Study	Sample Size	Calculated Blood Loss Mean (SD)	Change in Hb levels Means (SD)	Method of Measuring Blood Loss
Anh et al. 2022 [22]	739	NR	NR	Estimated
Cao et al. 2022 [21]	3180	NR	NR	Combination of estimated and quantitative
Chiruvolu et al. 2022 [22]	31	NR	NR	NR
Dayan-Schwartz et al. [23]	339	NR	1.4 (1.44)	Estimated
di Marco et al. 2023 [4]	707	NR	NR	Estimated
Drassinower et al. 2014 [24]	1009	NR	NR	Estimated
Huber et al. 2015 [25]	186	NR	NR	NR
Jiang et al. 2021 [26]	3212	NR	NR	NR
Kim et al. 2020 [27]	953	629.38 (187.48)	2.06 (1.24)	Estimated
Lin et al. 2021 [28]	288	NR	NR	Weighted
Lyu et al. 2022 [29]	2472	NR	NR	Weighted
Myles et al. 2001 [30]	19	NR	NR	Estimated
Santana et al. 2016 [31]	4756	NR	NR	Estimated
Sentilhes et al. 2022 [32]	319	1003.48 (1105.29)	1.65 (1.75)	Estimated
Seow et al. 2017 [33]	64	902.57 (384.21)	1.66 (1.45)	Estimated
Wenckus et al. 2014 [34]	2225	662.11 (292.40)	NR	Estimated
Ye et al. 2021 [35]	470	515.12 (379.94)	NR	Weighted
Yee at al. 2019 [36]	573	NR	NR	Estimated
Zhang et al. 2023 [37]	372	1350 (661.48)	NR	Weighted
Zhu et al. 2016 [38]	1071	NR	NR	NR
Zidan et al. 2022 [39]	345	NR	NR	Estimated

*Hb *Hemoglobin,* NR* Not Reported, *SD *Standard Deviation

### Obstetric outcomes

**Fig. 3:**
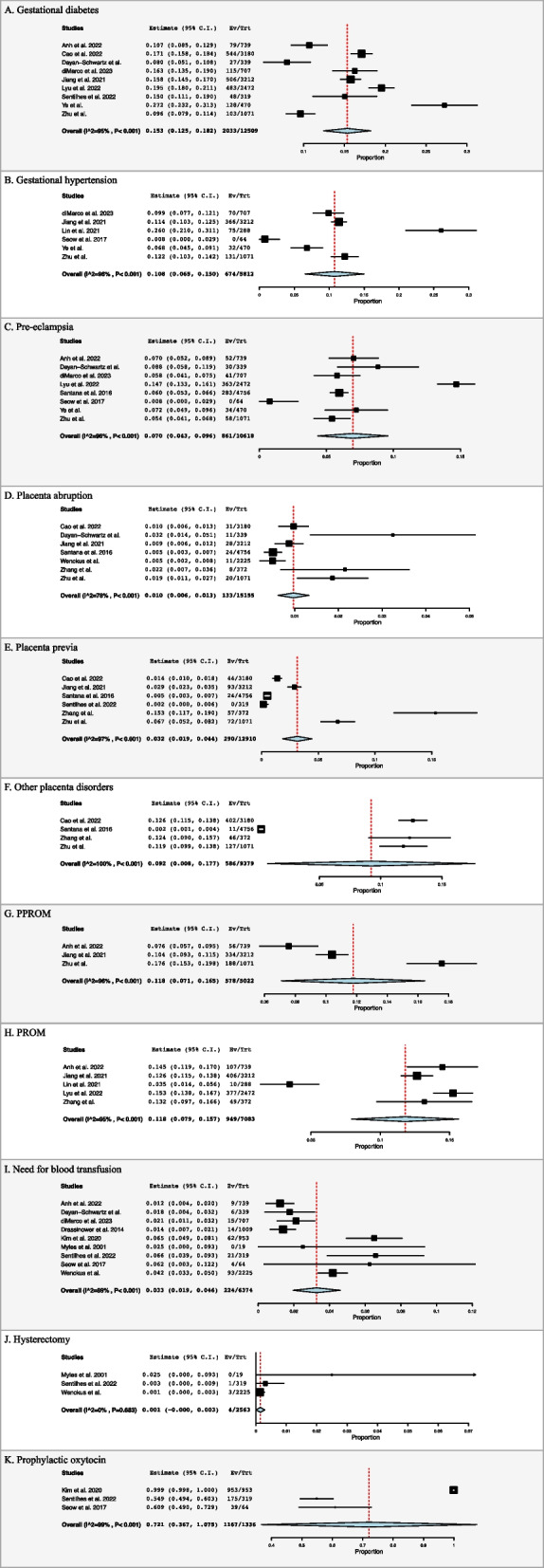
Forest plots for obstetric outcomes

On clinical assessment, prevalence of gestational diabetes was found to be 15.3% (95% CI: 0.125, 0.182, I^2^ = 95%, *n* = 2033), gestational hypertension was found to be 10.8% (95% CI: 0.065, 0.150, I^2^ = 96%, *n* = 674), and pre-eclampsia was found to be 7.0% (95% CI: 0.043, 0.096, I^2^ = 96%, *n* = 861) (Fig. 3A, B, and C**)** [4, 20, 21, 23, 26, 28, 29, 31–33, 35, 38].

Regarding placental disorders, prevalence of placental abruption, placenta previa, and those classified as other were 1.0% (95% CI: 0.006, 0.013, I^2^ = 78%, *n* = 133), 3.2% (95% CI: 0.019, 0.044, I^2^ = 97%, *n* = 290), 9.2% (95% CI: 0.008, 0.177, I^2^ = 100%, *n* = 586) respectively (Figs. 3D, E and F**)** [21, 23, 26, 31, 32, 34, 37, 38].

Prevalence of PPROM was 11.8% (95% CI: 0.071, 0.165, I^2^ = 96%, *n* = 578), while prevalence of PROM was 11.8% (95% CI: 0.079, 0.157, I^2^ = 95%, *n* = 949) (Figs. 3G and H**)** [20, 26, 28, 29, 37, 38].

Nine studies reported on the need for blood transfusion, which found prevalence to be 3.3% (95% CI: 0.019, 0.046, I^2^ = 89%, *n* = 224) (Fig. 3I) [4, 20, 23, 24, 27, 30, 32–34]. Three studies reported prevalence of post-partum hysterectomy to be 0.1% (95% CI: -0.000, 0.003, I^2^ = 0%, *n* = 4) (Fig. 3J) [30, 32, 34]. Four studies reported on change in haemoglobin with a mean decrease of 1.8 ± 1.4 (Table 2). Three studies reported on prophylactic oxytocin administration 72.1% (95% CI: 0.367, 1.075, I^2^ = 99%, *n* = 1167) (Fig. 3K) [27, 32, 33].

### Neonatal outcomes

**Fig. 4:**
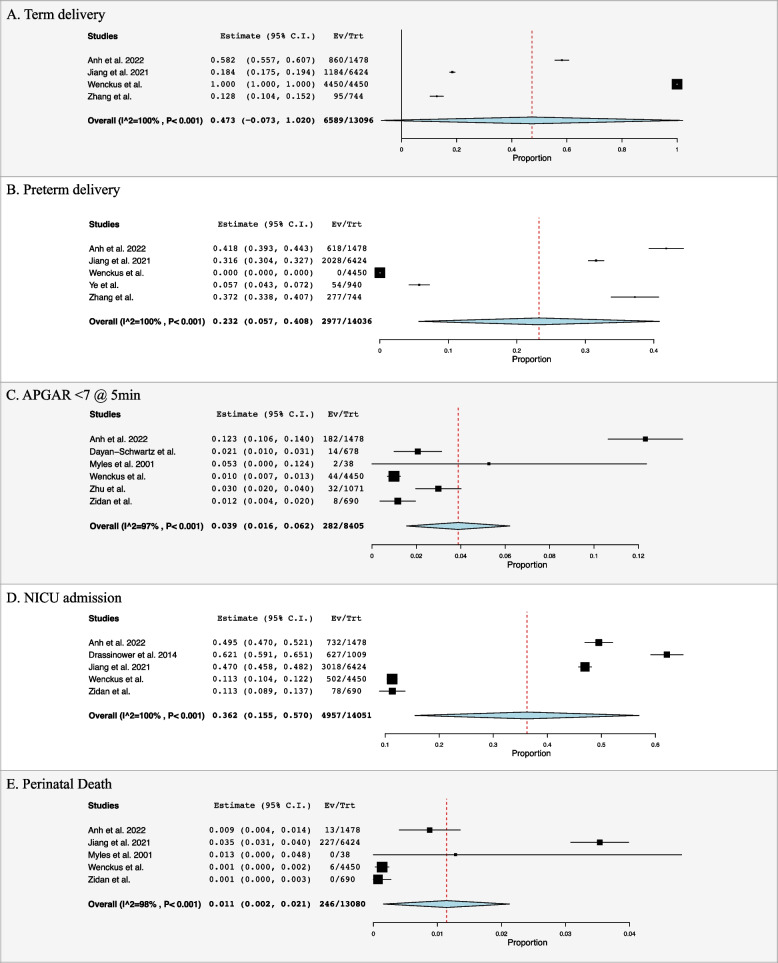
Forest plots for neonatal outcomes

Eleven studies reported birth weight with a mean weight of 2451.1 ± 499.0 g (Table 1) [4, 20, 22, 23, 26, 29, 30, 33–35, 39]. Prevalence of term deliveries and pre-term deliveries was found to be 47.3% (95% CI: -0.073, 1.020, I^2^ = 100%, *n* = 6589) and 23.2% (95% CI: 0.057, 0.408, I^2^ = 100%, *n* = 2977) respectively (Fig. 4A and B) [20, 26, 34, 35, 37]. Prevalence of APGAR scores < 7 at 5 min was 3.9% (95% CI: 0.016, 0.062, I^2^ = 97%, *n* = 282) (Fig. 4C) [20, 23, 30, 34, 38, 39]. Prevalence of NICU admissions was found to be 36.2% (95% CI: 0.155, 0.570, I^2^ = 100%, *n* = 4957) (Fig. 4D) [20, 24, 26, 34, 39]. The prevalence of perinatal death was found to be 1.1% (95% CI: 0.002, 0.021, I^2^ = 98%, *n* = 246) (Fig. 4E) [20, 26, 30, 34, 39].

## Discussion

### Main findings

Postpartum haemorrhage (PPH) is the most prevalent form of major obstetric haemorrhage and continues to be a significant contributor to maternal mortality rates in developed and developing countries. Whilst studied extensively in singleton pregnancies, corroborative literature in twin pregnancies remains lacking. Therefore, the primary aim of this systematic review and meta-analysis was to investigate the incidence of primary PPH among twin pregnancies whilst shedding light on maternal and peripartum characteristics. The incidence rate of PPH for vaginal deliveries reached 10.9% and for caesarean deliveries (CS), the rate soared to 27%. In vitro fertilization (IVF) was the most common conception method at 62% with 81.1% of twins being dichorionic diamniotic. During labor, 11.5% of mothers had a preterm pre-labor rupture of membranes (PPROM) whilst 13.4% had pre-labor rupture of membranes (PROM). Placental abruption was seen in 0.8% of pregnancies whilst placenta previa was seen in 2.2%. To the best of our knowledge, this meta-analysis is the first comprehensive collation on the current literature evidence on incidence rate of PPH in twin pregnancies. Highlighting this is crucial, as it provides vital information that can improve patient care, inform clinical guidelines, bolster patient education and counselling, and refine research and resource allocation efforts.

### Interpretation

Active management during third stage of labour in developed countries has reduced PPH incidence [40, 41]. But with rising twin pregnancies, especially in wealthier nations due to delayed childbearing, the risk increases [42–44]. Delayed childbearing often leads to increased use of assisted reproductive technology (ART), which raises the chance of multiple pregnancies, a known danger for maternal and fetal health [45]. Whilst single embryo transfer may mitigate risk, studies still show higher PPH risk in singleton pregnancies with ART [24] which is crucial given the global rise in twin pregnancies from ART [42, 44]. This is pertinent to the findings of the current meta-analysis demonstrating 62% of mothers using ART had IVF conception, suggesting infertility factors may contribute to PPH risk [23, 38].

Conditions like PCOS and endometriosis also increase risk, with PCOS also elevating chances of gestational diabetes, further complicating placental health [23, 38]. Furthermore, singleton ART pregnancies show higher rates of placental anomalies, including conditions like placenta previa and placenta accreta, which may be linked to PPH through disrupted placental attachment [46]. Although placental disorder rates in twins were low within this current meta-analysis, the increased uterine distention in twin pregnancies evidently raises PPH risk [47].

This is due to myometrial contractility compromise after delivery, subsequently leading to uterine atony [4]. Additionally, the increased weight of twins stretch the uterus myofibers to further exacerbate potential atony [48]. Twin pregnancy increases the placental bed area and its detachment exposes a large raw area potentially increasing the bleeding. Exacerbating factors such as iron deficiency and haemo-dilutional anaemia can compound the risk of PPH [7]. Loussert et al. observed that as the combined birth weight of twins increased, so did the risk of PPH, with a notable 88.8% rise observed for birth weights surpassing 6,500 g [27].

A commonly overlooked and undertreated risk factor in low-income regions that contribute to PPH is anaemia [41, 49, 50]. This stems from the impaired transport of haemoglobin and oxygen to the uterus compromising myometrial contractility [49]. Kavleh et al. found haemoglobin below 90 g/L increased blood loss during delivery and postpartum [50]. In our study, 5.25% of mothers were reported to have anaemia. However, this percentage may not fully capture the true prevalence of maternal anaemia due to the limited number of studies addressing this condition. Therefore, further research is warranted to better understand this potential association.

Routine use of active management of the third state of labour should be encouraged as data have shown how it significantly reduces PPH [40, 41]. Its key components include prompt administration of oxytocin following birth, controlled cord traction, and uterine massage post placental delivery [40, 41]. It has been highlighted that oxytocin may not be readily available or safe to use in low-income settings which is where misoprostol comes in as an alternative and effective uterotonic agent [40]. Tranexamic acid (TXA) has also garnered recognition for preventing and treating PPH, as shown in the WOMAN trial, where early administration reduced bleeding-related deaths by one-third. Prompt intervention within three hours of childbirth is most effective, per World Health Organisation (WHO) guidelines [51]. Accessibility of TXA in emergency obstetric care facilities warrants priority due to its cost-effectiveness and extended shelf life with multiple meta-analyses demonstrating reductions in blood loss, meaningful impactful in low-middle income countries and overall potential to reduce maternal mortality globally [51, 52].

In researching the challenges of addressing this topic, there exists no clear consensus on the definition of PPH. The Royal College of Obstetricians and Gynaecologists (RCOG) defines it as a blood loss of 500 mL or more, while the American College of Obstetricians and Gynecologists (ACOG) sets the threshold at 1000 mL, or blood loss accompanied by signs of hypovolemia [1, 41]. Ongoing discourse exists on whether early signs of hypovolemia should be included in the definition, especially considering increased blood volume during pregnancy, which can mask shock symptoms [1]. This is particularly relevant in low-income countries, where women are often severely anaemic before delivery. Unlike healthy women, severely anaemic women may not tolerate significant blood loss [1, 49, 50]. Additionally, neither RCOG nor ACOG's definitions account for differences in blood loss based on delivery method (vaginal versus CS). Within the current meta-analysis, the potential variations in blood loss depending on the method of delivery were accounted for. This nuance becomes crucial as the mode of delivery can also impact the risk and extent of PPH. Considering these factors is crucial for achieving a universally accepted definition and ensuring consistency in data and precision in managing PPH in twin pregnancies.

Furthermore, most included papers within this meta-analysis utilized a qualitative, estimate-based method for measuring blood loss, leading to discrepancies and underestimation. Visual assessment, for instance, can underestimate blood loss by 33–50% compared to photo-spectrometry [1]. While the gold standard is not practical for routine use, alternatives like calibrated drape bags offer improved precision, being 33% more accurate than visual estimation [1]. Using calibrated drapes not only enhances accuracy but also identifies PPH four times more often than visual estimation [1].

The current meta-analysis highlights that to further strengthen the evidence base and deliver more definitive conclusions, it is imperative to establish a universally agreed-upon definition and objective methods for measuring PPH. Obstetrician-gynecologists play a crucial role in informing patients about maternal reproductive risks, especially in cases of multiple pregnancies. Various research, in addition to the findings demonstrated in this paper, reveals a heightened risk of PPH among mothers carrying twins. This underscores the necessity for thorough discussions on potential complications and informed decision-making in reproductive choices [53].

### Limitations

While this meta-analysis presents novel insights, it is important to acknowledge its limitations. First, the lack of comparative two-arm analysis in the literature necessitated a single-arm approach. Consequently, we were unable to comprehensively analyze and draw definitive conclusions regarding the relationship between maternal and peripartum characteristics and the occurrence of PPH.

Secondly, the types of studies included in this paper present potential sources of bias. Given the nature of the topic, most of the included studies were retrospective and observational. This study design inherently exposes the analysis to several biases, such as selection bias and information bias, as well as confounding factors. These biases must be carefully considered when interpreting the outcomes of this meta-analysis. For instance, the limited number of included studies has contributed to a potential selection bias, as a high proportion of our population have undergone IVF. This bias may be linked to the increasing prevalence of twin pregnancies resulting from IVF, a trend driven by factors such as delayed childbearing and infertility [42, 44]. The rise in ART has not only increased the incidence of twin pregnancies but also spurred more research focusing on these cohorts. Consequently, the findings of this meta-analysis may be disproportionately influenced by IVF-related pregnancies, which could impact the generalizability of our results to the broader population of twin pregnancies. It is essential to recognize this limitation, as it underscores the need for future studies to include a more diverse range of twin pregnancies, both IVF and non-IVF, to provide a more comprehensive understanding of PPH risks in twin pregnancies. Moreover, many of our included studies were deemed high risk according to the ROBINS-I assessment. While this is an inherent limitation of the current meta-analysis, it nevertheless provides a foundation for future studies to thoroughly investigate the preliminary results presented in this study.

Thirdly, many of the included studies lacked the reporting of several secondary outcomes. Consequently, we were unable to perform a complete data extraction and analysis for the following outcomes: atony requiring uterotonics, uterine or hypogastric artery ligation, compression sutures, intrauterine balloon tamponade, exploratory laparotomy, and ICU admission. Larger cohort studies need to be conducted to address this gap.

Fourthly, factors such as varying demographic characteristics and differing approaches to measuring blood loss may have contributed to the high heterogeneity observed in the results. Furthermore, the single-arm nature of the analysis made it challenging to control for potential confounding factors, limiting the ability to draw definitive conclusions.

Fifthly, the absence of a consensus on the definition of PPH is a significant limitation. The definition we employed may have excluded numerous studies based on differing guidelines for PPH definitions, potentially leading to selection bias. Establishing a universally accepted definition is crucial not only for reducing bias in reporting and analysis but also for improving the management of PPH, to which this meta-analysis brings light to.

Lastly, the studies did not report on the wealth or income of the mothers, which prevented us from stratifying PPH risk by socioeconomic status. To further strengthen the evidence base and deliver more definitive conclusions, we recommend conducting more prospective studies with mothers matched for baseline characteristics to compare PPH rates.

## Conclusion

Our meta-analysis demonstrated that over one in ten vaginal deliveries may result in PPH, whilst this increases to over one in four in CS deliveries for twin pregnancies. IVF comprised the major form of conception within this patient population and appears to contribute to twin conception and subsequent PPH risk in selected mothers. As such, obstetric delivery teams must be adequately prepared for potential PPH in twin pregnancies. Although preliminary, the results of this meta-analysis provide evidence for the need to conduct further well-designed and high-quality studies to validate these findings.

## Supplementary Information

Supplementary Material 1.

Supplementary Material 2.

Supplementary Material 3.

## Data Availability

Data is provided within the data extraction excel sheet in the supplementary information files.

## Abbreviations

ACOG: American College of Obstetricians and Gynecologists
ART: Assisted Reproductive Technology
CS: Caesarean Delivery
IVF: In Vitro Fertilization
NICU: Neonatal Intensive Care Unit
PCOS: Polycystic Ovarian Syndrome
PPH: Post-Partum Haemorrhage
PPROM: Preterm Premature Rupture of Membranes
PROM: Premature Rupture of Membranes
RCOG: Royal College of Obstetricians and Gynaecologists
TXA: Tranexamic Acid
WHO: World Health Organization
