# Supplementary material for: Twin pregnancy and postpartum haemorrhage: a systematic review and meta-analysis
Source: BMC Pregnancy Childbirth. 2024 Oct 4;24:649. doi: 10.1186/s12884-024-06798-0 (PMC11451219; doi:10.1186/s12884-024-06798-0)
Supplement: Supplementary file 1 — Supplementary Material 1. [file 12884_2024_6798_MOESM1_ESM.docx]

Supplementary Item 1 - Twin Pregnancies Searches

PubMed 767

(twin* Pregnan* OR Twin Pregnancy OR "twins pregnant"[Title/Abstract:~4] OR "pregnancy, twin" OR "twin pregnancies" OR "twinning rate" OR "twin pregnancy" OR "pregnancy, multiple"[MeSH Terms] OR "multiple pregnancy"[All Fields] OR "multiple pregnancy"[Title/Abstract:~4] OR twin delivery OR "multiple birth pregnan*" OR "multiple pregnan*")

AND

('fluxus postpartum' OR 'haemorrhage, postpartum' OR 'hemorrhage, postpartum' OR 'post partum haemorrhage' OR 'post partum hemorrhage' OR 'postpartal haemorrhage' OR 'postpartal hemorrhage' OR 'postpartum bleeding' OR 'postpartum haemorrhage' OR 'puerperal haemorrhage' OR 'puerperal hemorrhage' OR 'postpartum hemorrhage' OR Postpartum Haemorrhage)

Which is:

(("twin*"[All Fields] AND "pregnan*"[All Fields]) OR ("pregnancy, twin"[MeSH Terms] OR ("pregnancy"[All Fields] AND "twin"[All Fields]) OR "twin pregnancy"[All Fields] OR ("twin"[All Fields] AND "pregnancy"[All Fields])) OR "twins pregnant"[Title/Abstract:~4] OR "pregnancy twin"[All Fields] OR "twin pregnancies"[All Fields] OR "twinning rate"[All Fields] OR "twin pregnancy"[All Fields] OR "pregnancy, multiple"[MeSH Terms] OR "multiple pregnancy"[All Fields] OR "multiple pregnancy"[Title/Abstract:~4] OR (("twins"[MeSH Terms] OR "twins"[All Fields] OR "twin"[All Fields]) AND ("deliveries"[All Fields] OR "delivery, obstetric"[MeSH Terms] OR ("delivery"[All Fields] AND "obstetric"[All Fields]) OR "obstetric delivery"[All Fields] OR "delivery"[All Fields])) OR ("multiple birth pregnan*"[All Fields] OR "multiple pregnan*"[All Fields])) AND (("fluxus"[All Fields] AND ("postpartum period"[MeSH Terms] OR ("postpartum"[All Fields] AND "period"[All Fields]) OR "postpartum period"[All Fields] OR "postpartum"[All Fields])) OR ("postpartum hemorrhage"[MeSH Terms] OR ("postpartum"[All Fields] AND "hemorrhage"[All Fields]) OR "postpartum hemorrhage"[All Fields] OR ("haemorrhage"[All Fields] AND "postpartum"[All Fields]) OR "haemorrhage postpartum"[All Fields]) OR ("postpartum hemorrhage"[MeSH Terms] OR ("postpartum"[All Fields] AND "hemorrhage"[All Fields]) OR "postpartum hemorrhage"[All Fields] OR ("hemorrhage"[All Fields] AND "postpartum"[All Fields]) OR "hemorrhage postpartum"[All Fields]) OR ("postpartum hemorrhage"[MeSH Terms] OR ("postpartum"[All Fields] AND "hemorrhage"[All Fields]) OR "postpartum hemorrhage"[All Fields] OR ("post"[All Fields] AND "partum"[All Fields] AND "haemorrhage"[All Fields]) OR "post partum haemorrhage"[All Fields]) OR ("postpartum hemorrhage"[MeSH Terms] OR ("postpartum"[All Fields] AND "hemorrhage"[All Fields]) OR "postpartum hemorrhage"[All Fields] OR ("post"[All Fields] AND "partum"[All Fields] AND "hemorrhage"[All Fields]) OR "post partum hemorrhage"[All Fields]) OR ("postpartal"[All Fields] AND ("blood"[MeSH Subheading] OR "blood"[All Fields] OR "blood"[MeSH Terms] OR "bloods"[All Fields] OR "haematology"[All Fields] OR "hematology"[MeSH Terms] OR "hematology"[All Fields] OR "haematoma"[All Fields] OR "hematoma"[MeSH Terms] OR "hematoma"[All Fields] OR "haemorrhage"[All Fields] OR "hemorrhage"[MeSH Terms] OR "hemorrhage"[All Fields] OR "haemorrhages"[All Fields] OR "hemorrhages"[All Fields] OR "haemorrhagic"[All Fields] OR "haemorrhaging"[All Fields] OR "hematologies"[All Fields] OR "haematomas"[All Fields] OR "hematomas"[All Fields] OR "hematoma s"[All Fields] OR "hematomae"[All Fields] OR "hemorrhaged"[All Fields] OR "hemorrhagic"[All Fields] OR "hemorrhagical"[All Fields] OR "hemorrhaging"[All Fields])) OR ("postpartal"[All Fields] AND ("blood"[MeSH Subheading] OR "blood"[All Fields] OR "blood"[MeSH Terms] OR "bloods"[All Fields] OR "haematology"[All Fields] OR "hematology"[MeSH Terms] OR "hematology"[All Fields] OR "haematoma"[All Fields] OR "hematoma"[MeSH Terms] OR "hematoma"[All Fields] OR "haemorrhage"[All Fields] OR "hemorrhage"[MeSH Terms] OR "hemorrhage"[All Fields] OR "haemorrhages"[All Fields] OR "hemorrhages"[All Fields] OR "haemorrhagic"[All Fields] OR "haemorrhaging"[All Fields] OR "hematologies"[All Fields] OR "haematomas"[All Fields] OR "hematomas"[All Fields] OR "hematoma s"[All Fields] OR "hematomae"[All Fields] OR "hemorrhaged"[All Fields] OR "hemorrhagic"[All Fields] OR "hemorrhagical"[All Fields] OR "hemorrhaging"[All Fields])) OR ("postpartum hemorrhage"[MeSH Terms] OR ("postpartum"[All Fields] AND "hemorrhage"[All Fields]) OR "postpartum hemorrhage"[All Fields] OR ("postpartum"[All Fields] AND "bleeding"[All Fields]) OR "postpartum bleeding"[All Fields]) OR ("postpartum haemorrhage"[All Fields] OR "postpartum hemorrhage"[MeSH Terms] OR ("postpartum"[All Fields] AND "hemorrhage"[All Fields]) OR "postpartum hemorrhage"[All Fields]) OR (("postpartum period"[MeSH Terms] OR ("postpartum"[All Fields] AND "period"[All Fields]) OR "postpartum period"[All Fields] OR "puerperal"[All Fields] OR "puerperally"[All Fields] OR "puerperant"[All Fields] OR "puerperants"[All Fields]) AND ("blood"[MeSH Subheading] OR "blood"[All Fields] OR "blood"[MeSH Terms] OR "bloods"[All Fields] OR "haematology"[All Fields] OR "hematology"[MeSH Terms] OR "hematology"[All Fields] OR "haematoma"[All Fields] OR "hematoma"[MeSH Terms] OR "hematoma"[All Fields] OR "haemorrhage"[All Fields] OR "hemorrhage"[MeSH Terms] OR "hemorrhage"[All Fields] OR "haemorrhages"[All Fields] OR "hemorrhages"[All Fields] OR "haemorrhagic"[All Fields] OR "haemorrhaging"[All Fields] OR "hematologies"[All Fields] OR "haematomas"[All Fields] OR "hematomas"[All Fields] OR "hematoma s"[All Fields] OR "hematomae"[All Fields] OR "hemorrhaged"[All Fields] OR "hemorrhagic"[All Fields] OR "hemorrhagical"[All Fields] OR "hemorrhaging"[All Fields])) OR (("postpartum period"[MeSH Terms] OR ("postpartum"[All Fields] AND "period"[All Fields]) OR "postpartum period"[All Fields] OR "puerperal"[All Fields] OR "puerperally"[All Fields] OR "puerperant"[All Fields] OR "puerperants"[All Fields]) AND ("blood"[MeSH Subheading] OR "blood"[All Fields] OR "blood"[MeSH Terms] OR "bloods"[All Fields] OR "haematology"[All Fields] OR "hematology"[MeSH Terms] OR "hematology"[All Fields] OR "haematoma"[All Fields] OR "hematoma"[MeSH Terms] OR "hematoma"[All Fields] OR "haemorrhage"[All Fields] OR "hemorrhage"[MeSH Terms] OR "hemorrhage"[All Fields] OR "haemorrhages"[All Fields] OR "hemorrhages"[All Fields] OR "haemorrhagic"[All Fields] OR "haemorrhaging"[All Fields] OR "hematologies"[All Fields] OR "haematomas"[All Fields] OR "hematomas"[All Fields] OR "hematoma s"[All Fields] OR "hematomae"[All Fields] OR "hemorrhaged"[All Fields] OR "hemorrhagic"[All Fields] OR "hemorrhagical"[All Fields] OR "hemorrhaging"[All Fields])) OR ("postpartum haemorrhage"[All Fields] OR "postpartum hemorrhage"[MeSH Terms] OR ("postpartum"[All Fields] AND "hemorrhage"[All Fields]) OR "postpartum hemorrhage"[All Fields]) OR ("postpartum haemorrhage"[All Fields] OR "postpartum hemorrhage"[MeSH Terms] OR ("postpartum"[All Fields] AND "hemorrhage"[All Fields]) OR "postpartum hemorrhage"[All Fields]))

EMBASE 1,049

Embase

Session Results

.......................................................

No. Query Results Results Date

#14. #9 AND #13 1,049 7 Jun 2023

#13. #10 OR #11 OR #12 21,794 7 Jun 2023

#12. ('post partum' OR postpartum) NEAR/4 (hemorrhage* 20,881 7 Jun 2023

OR haemorrage*)

#11. 'fluxus postpartum' OR 'haemorrhage, postpartum' 21,597 7 Jun 2023

OR 'hemorrhage, postpartum' OR 'post partum

haemorrhage' OR 'post partum hemorrhage' OR

'postpartal haemorrhage' OR 'postpartal

hemorrhage' OR 'postpartum bleeding' OR

'postpartum haemorrhage' OR 'puerperal

haemorrhage' OR 'puerperal hemorrhage' OR

'postpartum hemorrhage'

#10. 'postpartum hemorrhage'/exp 19,197 7 Jun 2023

#9. #1 OR #2 OR #3 OR #4 OR #5 OR #6 OR #7 OR #8 41,457 7 Jun 2023

#8. twin* NEAR/4 deliver* 4,131 7 Jun 2023

#7. 'twin delivery' 450 7 Jun 2023

#6. multiple NEAR/4 pregnan* 23,916 7 Jun 2023

#5. 'multiple birth pregnancies' OR 'multiple birth 20,212 7 Jun 2023

pregnancy' OR 'multiple pregnancies' OR

'pregnancy, multiple' OR 'multiple pregnancy'

#4. 'multiple pregnancy'/exp 30,118 7 Jun 2023

#3. twin* NEAR/4 pregnan* 20,039 7 Jun 2023

#2. 'pregnancy with twins' OR 'pregnancy, twin' OR 18,494 7 Jun 2023

'twin pregnancies' OR 'twinning rate' OR 'twin

pregnancy'

#1. 'twin pregnancy'/exp 14,404 7 Jun 2023

.......................................................

Cochrane 168

ID    Search      Hits

#1    MeSH descriptor: [Pregnancy, Twin] explode all trees  91

#2    MeSH descriptor: [Pregnancy, Multiple] explode all trees    320

#3    (twin* NEAR/4 pregnan*):ti,ab,kw (Word variations have been searched)   792

#4    ('pregnancy with twins' OR 'pregnancy, twin' OR 'twin pregnancies' OR 'twinning rate' OR 'twin pregnancy'):ti,ab,kw (Word variations have been searched)    1404

#5    ('multiple birth pregnancies' OR 'multiple birth pregnancy' OR 'multiple pregnancies' OR 'pregnancy, multiple' OR 'multiple pregnancy'):ti,ab,kw (Word variations have been searched)     5102

#6    (multiple NEAR/4 pregnan*):ti,ab,kw (Word variations have been searched)      1622

#7    ('twin delivery'):ti,ab,kw (Word variations have been searched)   606

#8    (twin* NEAR/4 deliver*):ti,ab,kw (Word variations have been searched)   189

#9    {OR #1-#8}  6267

#10   ('fluxus postpartum' OR 'haemorrhage, postpartum' OR 'hemorrhage, postpartum' OR 'post partum haemorrhage' OR 'post partum hemorrhage' OR 'postpartal haemorrhage' OR 'postpartal hemorrhage' OR 'postpartum bleeding' OR 'postpartum haemorrhage' OR 'puerperal haemorrhage' OR 'puerperal hemorrhage' OR 'postpartum hemorrhage'):ti,ab,kw (Word variations have been searched)   3165

#11   MeSH descriptor: [Postpartum Hemorrhage] explode all trees  963

#12   (('post partum' OR postpartum) NEAR/4 (hemorrhage* OR haemorrage*)):ti,ab,kw (Word variations have been searched) 2683

#13   #10 OR #11 OR #12 3453

#14   #9 AND #13  168

CiNAHL 141

| S14 | S9 AND S13 | Expanders - Apply equivalent subjects  Search modes - Boolean/Phrase | Interface - EBSCOhost Research Databases  Search Screen - Advanced Search  Database - CINAHL Complete | 141 |
| --- | --- | --- | --- | --- |
| S13 | S10 OR S11 OR S12 | Expanders - Apply equivalent subjects  Search modes - Boolean/Phrase | Interface - EBSCOhost Research Databases  Search Screen - Advanced Search  Database - CINAHL Complete | 5,831 |
| S12 | ('post partum' OR postpartum) N4 (hemorrhage* OR haemorrage*) | Expanders - Apply equivalent subjects  Search modes - Boolean/Phrase | Interface - EBSCOhost Research Databases  Search Screen - Advanced Search  Database - CINAHL Complete | 5,243 |
| S11 | 'fluxus postpartum' OR 'haemorrhage, postpartum' OR 'hemorrhage, postpartum' OR 'post partum haemorrhage' OR 'post partum hemorrhage' OR 'postpartal haemorrhage' OR 'postpartal hemorrhage' OR 'postpartum bleeding' OR 'postpartum haemorrhage' OR 'puerperal haemorrhage' OR 'puerperal hemorrhage' OR 'postpartum hemorrhage' | Expanders - Apply equivalent subjects  Search modes - Boolean/Phrase | Interface - EBSCOhost Research Databases  Search Screen - Advanced Search  Database - CINAHL Complete | 5,824 |
| S10 | (MM "Postpartum Hemorrhage") | Expanders - Apply equivalent subjects  Search modes - Boolean/Phrase | Interface - EBSCOhost Research Databases  Search Screen - Advanced Search  Database - CINAHL Complete | 2,827 |
| S9 | S1 OR S2 OR S3 OR S4 OR S5 OR S6 OR S7 OR S8 | Expanders - Apply equivalent subjects  Search modes - Boolean/Phrase | Interface - EBSCOhost Research Databases  Search Screen - Advanced Search  Database - CINAHL Complete | 8,296 |
| S8 | twin* N4 deliver* | Expanders - Apply equivalent subjects  Search modes - Boolean/Phrase | Interface - EBSCOhost Research Databases  Search Screen - Advanced Search  Database - CINAHL Complete | 967 |
| S7 | 'twin delivery' | Expanders - Apply equivalent subjects  Search modes - Boolean/Phrase | Interface - EBSCOhost Research Databases  Search Screen - Advanced Search  Database - CINAHL Complete | 782 |
| S6 | multiple N4 pregnan* | Expanders - Apply equivalent subjects  Search modes - Boolean/Phrase | Interface - EBSCOhost Research Databases  Search Screen - Advanced Search  Database - CINAHL Complete | 4,342 |
| S5 | 'multiple birth pregnancies' OR 'multiple birth pregnancy' OR 'multiple pregnancies' OR 'pregnancy, multiple' OR 'multiple pregnancy' | Expanders - Apply equivalent subjects  Search modes - Boolean/Phrase | Interface - EBSCOhost Research Databases  Search Screen - Advanced Search  Database - CINAHL Complete | 4,384 |
| S4 | twin* N4 pregnan* | Expanders - Apply equivalent subjects  Search modes - Boolean/Phrase | Interface - EBSCOhost Research Databases  Search Screen - Advanced Search  Database - CINAHL Complete | 3,859 |
| S3 | 'pregnancy with twins' OR 'pregnancy, twin' OR 'twin pregnancies' OR 'twinning rate' OR 'twin pregnancy' | Expanders - Apply equivalent subjects  Search modes - Boolean/Phrase | Interface - EBSCOhost Research Databases  Search Screen - Advanced Search  Database - CINAHL Complete | 4,066 |
| S2 | (MM "Pregnancy, Multiple+") | Expanders - Apply equivalent subjects  Search modes - Boolean/Phrase | Interface - EBSCOhost Research Databases  Search Screen - Advanced Search  Database - CINAHL Complete | 2,547 |
| S1 | (MM "Pregnancy, Twin") | Expanders - Apply equivalent subjects  Search modes - Boolean/Phrase | Interface - EBSCOhost Research Databases  Search Screen - Advanced Search  Database - CINAHL Complete | 1,094 |

Web of Science 658

# Searches:

1: TS=('pregnancy with twins' OR 'pregnancy, twin' OR 'twin pregnancies' OR 'twinning rate' OR 'twin

pregnancy') Date Run: Wed Jun 07 2023 14:47:57 GMT+1000 (Australian Eastern Standard Time) Results: 34601

2: TS=(twin* NEAR/4 pregnan*) Date Run: Wed Jun 07 2023 14:48:32 GMT+1000 (Australian Eastern Standard Time) Results: 10588

3: TS=('multiple birth pregnancies' OR 'multiple birth pregnancy' OR 'multiple pregnancies' OR 'pregnancy, multiple' OR 'multiple pregnancy') Date Run: Wed Jun 07 2023 14:49:36 GMT+1000 (Australian Eastern Standard Time) Results: 36464

4: TS=(multiple NEAR/4 pregnan*) Date Run: Wed Jun 07 2023 14:50:18 GMT+1000 (Australian Eastern Standard Time) Results: 9256

5: TS=('twin delivery') Date Run: Wed Jun 07 2023 14:50:53 GMT+1000 (Australian Eastern Standard Time) Results: 6969

6: TS=(twin* NEAR/4 deliver*) Date Run: Wed Jun 07 2023 14:51:49 GMT+1000 (Australian Eastern Standard Time) Results: 2667

7: #6 OR #5 OR #4 OR #3 OR #2 OR #1 Date Run: Wed Jun 07 2023 14:52:28 GMT+1000 (Australian Eastern Standard Time) Results: 69414

8: TS=('fluxus postpartum' OR 'haemorrhage, postpartum' OR 'hemorrhage, postpartum' OR 'postpartum haemorrhage' OR 'post partum hemorrhage' OR 'postpartal haemorrhage' OR 'postpartal hemorrhage' OR 'postpartum bleeding' OR 'postpartum haemorrhage' OR 'puerperal haemorrhage' OR 'puerperal hemorrhage' OR 'postpartum hemorrhage') Date Run: Wed Jun 07 2023 14:55:07 GMT+1000 (Australian Eastern Standard Time) Results: 12348

9: TS=('post partum' NEAR/4 hemorrhage*) Date Run: Wed Jun 07 2023 15:02:26 GMT+1000 (Australian Eastern Standard Time) Results: 654

10: TS=('post partum' NEAR/4 haemorrhage*) Date Run: Wed Jun 07 2023 15:02:52 GMT+1000 (Australian Eastern Standard Time) Results: 598

11: TS=('postpartum' NEAR/4 haemorrhage*) Date Run: Wed Jun 07 2023 15:04:19 GMT+1000 (Australian Eastern Standard Time) Results: 2301

12: TS=('postpartum' NEAR/4 hemorrhage*) Date Run: Wed Jun 07 2023 15:04:41 GMT+1000 (Australian Eastern Standard Time) Results: 7099

13: #8 OR #9 OR #10 OR #11 OR #12 Date Run: Wed Jun 07 2023 15:05:24 GMT+1000 (Australian Eastern Standard Time) Results: 12349

14: #13 AND #7 Date Run: Wed Jun 07 2023 15:06:02 GMT+1000 (Australian Eastern Standard Time) Results: 658

Scopus 905

**( ( TITLE-ABS-KEY ( *"pregnancy with twins"*  OR  *"pregnancy, twin"*  OR  *"twin pregnancies"*  OR  *"twinning rate"*  OR  *"twin pregnancy"* ) )  OR  ( TITLE-ABS-KEY ( *twin**  W/4  *pregnan** ) )  OR  ( TITLE-ABS-KEY ( *"multiple birth pregnancies"*  OR  *"multiple birth pregnancy"*  OR  *"multiple pregnancies"*  OR  *"pregnancy, multiple"*  OR  *"multiple pregnancy"* ) )  OR  ( TITLE-ABS-KEY ( *multiple*  W/4  *pregnan** ) )  OR  ( TITLE-ABS-KEY ( *"twin delivery"* ) )  OR  ( TITLE-ABS-KEY ( *twin**  W/4  *deliver** ) ) )**

**AND**

**( ( TITLE-ABS-KEY ( *"fluxus postpartum"*  OR  *"post partum haemorrhage"*  OR  *"post partum hemorrhage"*  OR  *"postpartal haemorrhage"*  OR  *"postpartal hemorrhage"*  OR  *"postpartum bleeding"*  OR  *"postpartum haemorrhage"*  OR  *"puerperal haemorrhage"*  OR  *"puerperal hemorrhage"*  OR  *"postpartum hemorrhage"*  OR  *postpartum*  AND *haemorrhage* ) )  OR  ( TITLE-ABS-KEY ( ( *"post partum"*  OR  *postpartum* )  W/4  ( *hemorrhage**  OR  *haemorrhage** ) ) ) )**
